# The Cost of Suspected and Confirmed Bacterial Meningitis Cases Treated at Jimma University Medical Center, Ethiopia

**DOI:** 10.4314/ejhs.v32i4.13

**Published:** 2022-07

**Authors:** Temesgen Kabeta Chala, Teferi Daba Lemma, Kora Tushune Godana, Melkamu Berhane Arefayine, Alemseged Abdissa, Esayas Kebede Gudina

**Affiliations:** 1 Department of Health Policy and Management, Institute of health, Jimma University, Jimma, Ethiopia; 2 Department of Pediatrics and Child Health, Institute of Health, Jimma University, Jimma, Ethiopia; 3 Armauer Hansen Research Institute, Addis Ababa, Ethiopia; 4 Department of Internal Medicine, Institute of Health, Jimma University, Jimma, Ethiopia

**Keywords:** Meningitis, suspected bacterial meningitis, confirmed bacterial meningitis, cost of admission

## Abstract

**Background:**

Infections of the central nervous system (CNS) such as meningitis or encephalitis can be caused by myriad of microorganisms and may be life-threatening. In Ethiopia, it is an important cause of premature death and disability, being the 9^th^ most common cause of years of life lost and loss of disability-adjusted life years.

The objective of this study was to estimate the cost of suspected and confirmed bacterial meningitis among inpatient managed patients at JUMC.

**Methods:**

A facility-based cross-sectional study was conducted from July 28 to September 12, 2018. A semi-structured questionnaire was used in this study. Checklists were used to collect the types of laboratory tests performed and prescribed medications. This cost of illness study was conducted from the patient perspectives. We employed a micro-costing bottom-up approach to estimate the direct cost of meningitis. The human capital approach was used for estimating wages lost.

**Result:**

Among total patients admitted and treated in JUMC, higher proportions (69.8%) were suspected bacterial meningitis but have been treated as confirmed cases. Total median costs for both suspected and confirmed bacterial meningitis patients were estimated to be ETB 98,812.32 (US $ 3,593.2; IQR 1,303.0 to 5,734.0). Total median direct cost was ETB 79,248.02 (US $ 2,881.75; IQR 890.7 to 3,576.7). Moreover, 45.3% of the patients reported that they were either admitted or given medication at JUMC or nearby health facility before their current admissions.

**Conclusion:**

These findings indicate that most cases of bacterial meningitis were treated only empirically, and the cost of the treatment was high, especially for resource-limited countries like Ethiopia. To minimize the burden of meningitis and avoid unnecessary hospitalizations, the availability of diagnostic techniques is vitally important.

## Introduction

Central nervous system (CNS) infections involving the brain (cerebrum and cerebellum), spinal cord, optic nerves, and their covering membranes are medical emergencies that are associated with substantial morbidity, mortality, or long-term sequelae that may have catastrophic implications for the quality of life of affected individuals (1).

Despite advances in clinical care, bacterial meningitis remains a severe disease with a high risk of complications that may lead to death or severe sequelae which could be very high in meningitis belt of Africa (2). The meningitis belt of sub-Saharan Africa runs across the continent from Senegal to Ethiopia. This makes the regions prone to major epidemics of meningococcal meningitis(3).

Lumbar puncture and analysis of cerebrospinal fluid may be done primarily to exclude bacterial meningitis, but identification of the specific viral cause is beneficial (4). Due to similarities in symptomatology, the identification of a specific viral agent in body fluids, especially the CSF, in a patient with aseptic meningitis is of more than academic interest since it can shorten the duration of hospital stay and eliminate unnecessary antimicrobial therapy (4).

Fatal complications due to the primary infection with meningitis is most common within 14 days of admission. The diversity of complications causing death in meningitis suggests that determining the clinical cause of death is essential to the evaluation of novel treatment strategies (5). Meningitis has also a significant influence on mortality, morbidity, education, and income (6).

Prompt initiation of antibiotics in patients suspected to have bacterial meningitis is essential to improve the overall outcome of patients. However, subsequent management of these patients should be guided by findings of CSF analysis and alternative diagnoses should be considered in the absence of supportive evidence for acute bacterial meningitis (7).

A careful clinical judgment is mandatory to keep the balance of proper pre-emptive management of ABM and avoid mis-diagnosis of other CNS conditions (7). Increasing bacterial antimicrobial resistance has prompted physicians to choose broad-spectrum antimicrobials in order to reduce the likelihood of inactive empirical therapy(8). The World Health Organization (WHO) argued for pathways to universal health care coverage (UHC) accounting for the reduction of out-of-pocket direct payments and financial risk protection as one essential dimension to measure progress on the way to UHC (9).

Likewise, the Sustainable Development Goal for health (SDG3) included a sub-target on achieving “universal health coverage,” including financial risk protection and access to quality essentials (10).

This study aimed to estimate both the direct and indirect costs associated with the treatment of presumed bacterial meningitis at a tertiary hospital in Ethiopia.

## Method and Materials

This study was conducted at Jimma University Medical CenterMedical center (JUMC). JUMC is one of the oldest public hospitals in Ethiopia, which is located in Jimma town 346.1 km southwest of Addis Ababa. The town has a population of 207,573 according to the 2012 census. There are six public health institutions (four health centers & five hospitals, among which three were private hospitals) in the town.

JUMC is the only teaching and referral hospital in southwestern Ethiopia with a catchment population of about 20 million people. A facility-based cross-sectional study was conducted from July 28 to September 12, 2018.Participants of the study were all patients (both pediatrics and adults) treated at JUMC for suspected or confirmed bacterial meningitis during the study period. A semi-structured questionnaire was used to collect sociodemographic and clinical characteristics of the patients.

Checklists were used to collect types of laboratory tests performed and prescribed medications. Data on direct medical costs (registration/consultation, diagnostic workup, medications, and hospital bed), direct non-medical costs (transportation, food, and drinks, lodging) and caregivers'/parents'/friends'work time loss was collected through interviews.

This cost of illness study was conducted from the patient perspectives. It employed a microcosting bottom-up approach to estimate the direct cost of meningitis. Indirect costs for the study subjects and their accompanying persons were calculated in terms of wage losses using the human capital approach. Application of the Human Capital Approach (HCA) was applied as per the cost-based approach (based on education costs). This measures the costs of education and the university system based on actual expenditure (resources used for the training of human capital) and opportunity costs (time devoted to education, missed student earnings (11).

For our analysis, the cost of meningitis care was defined as the sum of direct medical, direct non-medical, and indirect costs. The indirect costs were estimated earnings lost because of travel to and from hospitals, hospitalization and absences from work because of illness related to meningitis patients and their families. These workdays were converted into monetary terms using the human capital approach for skilled respondents and the minimum wage for unemployed respondents.

Out of pocket expenses were measured in terms of Ethiopian Birr (ETB) and converted to US dollars (US$). The mean of August and September 2018 exchange rates of 1 US dollar for

**27.50** ETB were used to convert ETB to US dollars (12). The inflation rate calculated for the 2018 base year was considered during comparison with other studies. The Ethiopian inflation rate was 13.0% in 2018 (13).

**Figure 1:**
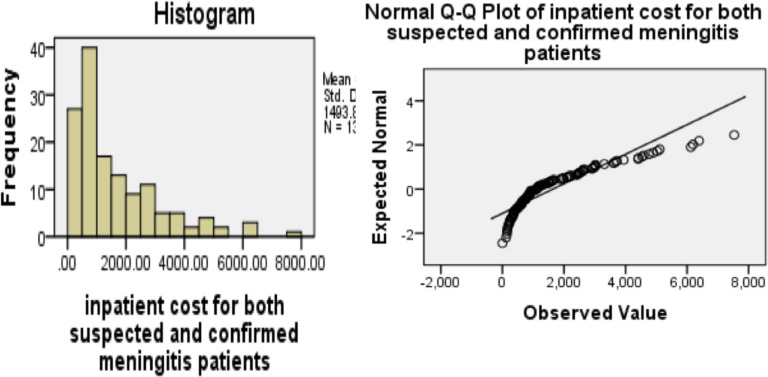
Cost distribution of suspected and confirmed bacterial meningitis at JUMC Jimma, Southwest Ethiopia, June to September 2018 (1 US $ = 27.50 ETB).

In the tests of normality, the results of the Kolmogorov-Smirnov and Shapiro-Wilk tests indicate that the distribution remains significantly different from a normal distribution at P < 0.0001. Since the data was skewed to the right or positively skewed, we used non-parametric tests to compare the median values. The Mann-Whitney test for variables that have only two independent groups and the Kruskal Wallis test for variables with more than two independent groups were used to compare the cost of suspected and confirmed bacterial meningitis (Figure 1).

Diagnosis, sex, residence, previous admissions, and marital status were considered for Mann-Whitney tests. The Kruskal-Wallis test was used for distance from JUMC, income, age, family size, and occupation.

To ensure data quality assurance, we have provided two days of training for data collectors and supervisors. The questionnaire was translated into Afan Oromo and Amharic languages and back-translated to English to check for consistency using language experts.

A pre-test of the questionnaire was conducted among seven (5%) patients admitted to a different hospital (Shenen Gibe Hospital) on all admissions, and we have made minor modifications based on our pre-test result. The collected data was cleaned, coded, and entered into Epi Data 3.1 using double data entry and exported to SPSS version 20 for analysis, and the result was presented using tables, graphs, and text. Demographic information was described by frequency and proportion.

Ethical clearance was secured from Jimma University's Institutional Review Board (IRB) before data collection. A permission letter was obtained from the school of graduate studies and submitted to JUMC. Written consent was obtained from our study participants. In addition, voluntary participation, anonymity, confidentiality, and privacy were ensured. Families and caregivers have consented for children.

## Results

**Description of study participants**: Our study included 139 patients with suspected or confirmed bacterial meningitis. Most of the patients, 106 (76.3%) were treat with out-of-pocket payment. The treatment costs for the rest, 21.5% and 2.2%, were covered by community-based health insurance and missionary charity respectively.

We surveyed all inpatients with suspected and confirmed bacterial meningitis in all wards during the data collection period. Neonates and children contributed for 85 (61.2%) of the participants while the rest were recruited from medical wards. Higher proportions (69.8%) were treated as suspected bacterial meningitis. However, they were treated with the same regimen as confirmed bacterial meningitis.

More than half of the study participants, 79 (56.8%) were males (Table 1).

**Table 1:** Demographic and socioeconomic characteristics of patients treated as bacterial meniningitis at JUMC, Jimma, Southwest Ethiopia, July to September 2018

Variables	Frequency	%
Age (years)		
Less than 4	99	71.2
5–15	19	13.7
>15	21	15.1
Educational level		
Under school age and illiterate	104	74.8
Read and write only	5	3.6
Grade 1–8	21	15.1
Grade 9–12	6	4.3
College/university	3	2.2
Distance from JUMC		
Less than 50 km	71	51.1
50–100 km	32	23.0
100–300 km	25	18.0
Greater than 301	10	7.2
Family size		
1–2	1	0.7
3–5	75	54.0
6–8	56	40.3
9–11	6	4.3
11+	1	0.7
Diagnosis		
Suspected bacterial meningitis	97	69.8
Confirmed bacterial meningitis	42	30.2
Inpatient admission days		
Less than 5 days	17	12.2
6–10 days	47	33.8
11–15 days	41	29.5
> 16 days	34	24.5
**Marital status**		
Single	118	84.9
Married	21	15.1
**Permanent residence**		
Out of Jimma	82	59.0
Jimma	57	41.0
**Accompanying person**		
No	25	18.0
Yes	114	82.0
Sex		
Female	60	43.2
Male	79	56.8
**Monthly income in ETB (n=118)**		
1–500	4	2.9
501–1000	13	9.4
1001–1500	8	5.8
1501–2000	9	6.5
2000–5000	53	38.1
5000 +	26	18.7

**Pre-admission costs prior to current JUMC admissions**: We asked respondents if they had been admitted and treated for any health problem prior to current admissions. Accordingly, 63 (45.3%) of respondents had responded that they had been admitted and treated at JUMC or nearby health facilities for at least a day with the total median cost of ETB 2500.85 (US $ 90.94; IQR 12 to 155) before current admissions.

The median time used to seek care for the study participants was 2 h 50 min (2:50). The median total direct non-medical cost (DNMC) was ETB 4587 (US$ 166.8; IQR 65.7 to 288.6) for suspected and confirmed bacterial meningitis.

**Patient's side costs at JUMC**: The median total costs for both suspected and confirmed bacterial meningitis were estimated to be ETB 98,812.32 (US $ 3,593.2; IQR ***1,303.0 to 5,734.0***).

**Direct cost**: The total median direct cost of both suspected and confirmed bacterial meningitis was ETB 79,248.02 (US $ 2,881.75; IQR 2,103.1–8,765.3). Direct costs were classified as direct medical and direct non-medical costs. A higher proportion, 80.2% (US $ 2,311.2; IQR, 650.4 to 4.987.0), of the direct costs were direct medical costs.

**Indirect costs**: The median estimated total indirect cost was ETB 19,564.83 (US $ 711.4; IQR, 210.0 to 1,670.2) for both patients and companions. A higher proportion (79 %) of all companions responded that they lost income due to the hospitalization of the patients. A median of six working days (IQR 0 to 11) were lost due to the current admisions days. The overall cost of treatment was found to be higher among suspected bacterial menigitis as compared with confirmed cases (P = 0. 016). A significant difference in median costs was also observed by income group (P = 0.025) and family size (P = 0.008). Patients who came from families of more than five had greater costs than those with lower family sizes (Table 2).

**Table 2:** Median and interquartile range for patient-side costs for suspected and confirmed bacterial meningitis at JUMC, Jimma, Southwest Ethiopia, July–September 2018.(1US $= 27.50 ETB) (Mann-Whitney and Kruskal-Wallis Test, p-value)

Variables	Median (ETB)	IQR	P-value
**Diagnosis**			
Confirmed bacterial meningitis	5,130.3	(2,103.1–8,765.3)	0.016*
Suspected bacterial meningitis	5,863.41	(2,300.2–9,78.5)	
**Sex**			
Male	6,161.08	(2,609.0–1108.7)	0.233
Female	4,985.19	(1,890.4–6,540.5)	
**Residence**			
Out of jimma	5,806.83	(2,104.0–8,908.1)	0.37
Jimma	5,432.91	(1,998.4–7,035.8)	
**Cost of previous admission**			
Yes	27,512.1	(10,345.6–87,678.9)	0.16
No	-		
**Accompanied**			
Yes	5,739.72	(2,567.8–10,342.1)	0.922
No	5,220.28	(1,700.9–6,903.2)	
**Marital status**			
Single	5,027.18	(1,856.9–7022.4)	0.075
Married	9,174.19	(3045.7–16,765.0)	
**Distance from JUMC (KM)**			
Less than 50 km	4704.32	(1,845.9–6457.8)	
51–100 km	5425.66	(2,105.7–6,945.6)	0.184
101–300km	5602.36	(2,563.2–8970.1)	
Greater than 301km	13,072.80	(3,400.0–23,456.0)	
**Income**			
less than 500	2870.25	(890.0–5402.1))	
501–2000	4339.67	(1675.4–6342.1)	0.025*
2001–4000	7881.44	(2700.8–15587.0)	
4000+	5157.50	(2043.1–6878.9)	
**Age**			
under 4	5543.22	(2598.0–8890.0)	0.407
5–15	6122.16	(2678.9–11,234.0)	
16–50	5685.90	(2,763.2–9080.1)	
**Family size**			
1–2	-	-	
3–5	4528.61	(1775.4–7342.1)	**0.008** *
6–8	5501.13	(2498.0–8870.0)	
9–11	7786.67	((2790.8–15987.0))	
11+			
**Occupation**			
Farmer	9991.50	(4300.4–17,650.0)	
House Wife	1317.50	(230.0–3,789.4)	
Government Employed	12207.63	(198.8–3567.0)	0.261
Non-governmental organization	5538.00	(2498.0–8790.0)	
Private	6195.00	(2688.9–10,234.0)	
Merchant	5553.00	(2498.0–8690.0	
Unemployed	5046.08	(1598.0–7890.0	

* Significant at 95 % level of confidence

There was no significant difference in the median cost by age and sex group, current residence, previous admission, marital status, occupation, and patient's residence distance from (Table 2).

## Discussion

This study revealed that the median total cost for suspected and confirmed bacterial meningitis was ETB 98,812.32 (US $ 3593.2). The median cost of suspected and confirmed bacterial meningitis was significantly different with the family size of respondents. This could be because Ethiopians are a known culture of companionship when a family member or neighbor is sick and hospitalized.

Our finding is high when compared to studies conducted in Ghana which was US $ 201.7; corrected for inflation US $ 225.5 (14), Senegal US $90; corrected for inflation US $91.89 (15), and Pakistan US $ 235; corrected for inflation US $ 244.6 (16) among patients admitted to the district hospitals.

The higher costs could be due to admitting and treating suspected bacterial meningitis as confirmed due to the lack of accurate diagnostic workup to differentiate the etiologies of meningitis in Ethiopia. Our costing method, the difference in diagnostic technology, health facilities set up, the pattern of out-of-pocket payments, and the difference in health care financing could have been also contributed to such a significant difference.

The total median direct cost of both suspected and confirmed bacterial meningitis was ETB 79,248.02 (US $ 2881.75). This finding is close to the finding in Vietnam (17). This similarity might be due to the similarity in the average length of hospital stay of 13 days in Vietnam and the cost variables included were similar for both studies. 80.2% was direct medical cost (DMC), while only 19.8% was direct non-medical cost (DNMC). More than half (53.3%) of DMC was the cost of drugs, and the cost of investigation accounted for 46.7% of the cost of direct medical costs.

This was supported by the study in Vietnam in which the cost of drug contributed for 40–60 % of DMC (17). This similarity might be because of drug costs across the world and the pattern of treatment similarities for meningitis.

Higher proportions (69.8%) of those diagnosed as suspected bacterial meningitis who were treated as confirmed bacterial meningitis might have contributed to the increase in the total costs. In JUMC, even though patients were negative for bacterial meningitis using the current standard of diagnosis, they were given full antibiotic regimen in fear of a false-negative diagnosis arising from self-medication.

The method of diagnosis of bacterial meningitis in JUMC (culture and gram stain) is highly affected by previous antibiotics

The study conducted in teaching hospitals in Ethiopia, identified most patients who were treated for suspected BM did not receive a proper diagnostic workup and were treated only based on clinical suspicion (7).

According to a study conducted in the USA, hospitalization rates for viral meningitis remain high due to the concern for bacterial meningitis, a severe condition that presents with similar initial symptoms (headache, fever, and irritability) and requires immediate treatment with parenteral antibiotics (18).

Because bacterial cultures may take days to grow, treatment decisions must be made before definitive results are available (7). Such situations are prevalent in resource-limited countries like Ethiopia.

From these arguments, we can conclude that more than half of the patients who were on antibacterial treatment at JUMC during the study period might have had viral meningitis. Antibiotics are not effective against viruses, although, in some instances, antibiotics may be started on admission to the hospital because the cause of meningitis is not known. Considering an accurate method of diagnosis and differentiation, in addition to minimizing unnecessary admissions, an estimated US $2,867.37 could be saved at an average length of hospital stay of 11 days. This amount is approximately equal to the annual salary of a new graduate bachelor's degree health professional in Ethiopia.

The limitations of this study include selection bias (only patients treated at tertiary referral hospital), information bias (memory loss), being unable to measure intangible costs, and factors associated with direct costs were not assessed. We conducted the cost estimation only from the patient's perspective, which could lead to overestimations.

In an attempt to reduce the effects of the above limitations, patients and companions were given enough time to recall costs, information was cross-checked from patients' charts, and the result of the finding was generalized only to patients with suspected and confirmed bacterial meningitis.

In conclusion, this finding indicates that the cost of meningitis is high, especially for resourcelimited countries like Ethiopia. Direct and indirect costs inflict that meningitis is a serious health problem, demanding the attention of health program managers, planners, and policy designers. So, a higher proportion of suspected cases could be because of previous medications as culture (the current gold standard diagnosis) results are affected grossly by previous antibiotics. Additionally, the current diagnostic approach (bacterial culture) cannot identify viral meningitis from the others.

Thus, to minimize the burden of bacterial meningitis, and avoid unnecessary hospitalizations expenses, the availability of diagnostic techniques is essential.
